# The fumarate salts of the *N*-isopropyl-*N*-methyl derivatives of DMT and psilocin

**DOI:** 10.1107/S2056989019011253

**Published:** 2019-08-16

**Authors:** Andrew R. Chadeayne, Duyen N. K. Pham, James A. Golen, David R. Manke

**Affiliations:** a CaamTech, LLC, 58 East Sunset Way, Suite 209, Issaquah, WA 98027, USA; b 285 Old Westport Rd., North Dartmouth, MA, 02747, USA

**Keywords:** crystal structure, tryptamines, hydrogen bonding

## Abstract

The solid-state structures of the salts of two substituted tryptamines, namely *N*-isopropyl-*N*-methyl­tryptaminium (MiPT) fumarate {systematic name: [2-(1*H*-indol-3-yl)eth­yl](meth­yl)propan-2-yl­aza­nium 3-carb­oxy­prop-2-enoate}, C_14_H_21_N_2_^+^·C_4_H_3_O_4_^−^, and 4-hy­droxy-*N*-isopropyl-*N*-methyl­tryptaminium (4-HO-MiPT) fumarate monohydrate {systematic name: [2-(4-hy­droxy-1*H*-indol-3-yl)eth­yl](meth­yl)propan-2-yl­aza­nium 3-carb­oxy­prop-2-enoate monohydrate}, C_14_H_21_N_2_O^+^·C_4_H_3_O_4_^−^·H_2_O, are reported. Both salts possess a proton­ated tryptammonium cation and a 3-carb­oxy­acrylate (hydrogen fumarate) anion in the asymmetric unit; the 4-HO-MiPT structure also contains a water mol­ecule of crystallization. Both cations feature disorder of the side chain over two orientations, in a 0.630 (3):0.370 (3) ratio for MiPT and a 0.775 (5):0.225 (5) ratio for 4-HO-MiPT. In both extended structures, N—H⋯O and O—H⋯O hydrogen bonds generate infinite two-dimensional networks.

## Abstract 2

The crystal structures of the fumarate salts of the psychomimetics MiPT and 4-HO-MiPT are reported. The extended structure of both compounds feature two-dimensional networks of ions connected through N—H⋯O and O—H⋯O hydrogen bonds.

## Chemical context   

*N*,*N*-di­methyl­tryptamine (DMT) and its derivatives have been used by humans for centuries because of their psychoactive, entheogenic, or hallucinogenic effects, or combinations thereof (Cameron & Olson, 2018 ▸). Psilocybin, the 4-phosphate variant of DMT, is arguably its most studied derivative. Psilocybin is one of several naturally occurring psychoactive tryptamines found in ‘magic’ mushrooms. When consumed by humans, psilocybin serves as a prodrug of psilocin. Upon digestion, psilocybin hydrolyses to generate psilocin, the 4-hy­droxy derivative of DMT. Psilocin is a potent seratonin 2a-agonist, which is responsible for its psychoactive properties (Dinis-Oliveira, 2017 ▸; Nichols, 2012 ▸). Psychoactive tryptamines like DMT and psilocin have garnered significant inter­est recently because of their potential for treating mood disorders, including depression, anxiety, addiction, and post-traumatic stress disorder (PTSD) (Johnson & Griffiths, 2017 ▸; Carhart-Harris & Goodwin, 2017 ▸).

Altering the chemical structure within this class of compounds can dramatically influence the potency and action of the drugs. For example, merely changing the *N*,*N*-dialkyl groups on DMT can modify its psychoactive properties: increasing the chain length of the two alkyl groups of the tryptamine to larger than *n*-butyl dramatically reduces or eliminates the psychoactive effects (Bradley & Johnston, 1970 ▸).

The synthesis of *N*-methyl-*N*-iso­propyl­tryptamine (MiPT) was reported in 1981 (Repke *et al.*, 1981 ▸). In 1985, Repke and co-workers reported that of the compounds in the series of *N*,*N*-dialkyl-4-hy­droxy­tryptamines, the *N*-methyl-*N*-isopropyl derivative (4-HO-MiPT) is the most potent based upon qualitative effects on humans (Repke *et al.*, 1985 ▸). Later qu­an­ti­tative studies showed the *N*-methyl-*N*-isopropyl deriv­atives of DMT and psilocin to be more potent as seratonin-1A, −2A and −2B receptors compared to the analogous dimethyl compounds (McKenna *et al.*, 1990 ▸).
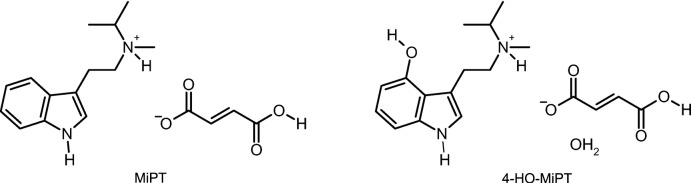


Improving our understanding of how these drugs inter­act with particular biological receptors requires a complete understanding of their chemical structures. Given their therapeutic potential and the significant structure–activity relationship between them, further studies would benefit from better understanding of their chemical structures. Responding to this unmet need, we report the crystal structures of the fumarate salts of MiPT and 4-HO-MiPT herein.

## Structural commentary   

**Figure 1:**
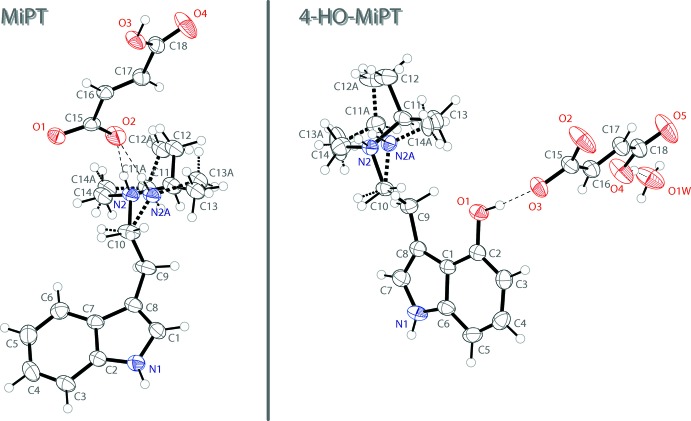
The mol­ecular structure of MiPT fumarate (left) and HO-MiPT fumarate (right), showing the atomic labeling. Displacement ellipsoids are drawn at the 50% probability level. Dashed bonds indicate a disordered component in the structures. Hydrogen bonds are shown as dashed lines.

The mol­ecular structure of MiPT fumarate is shown on the left of Fig. 1 ▸. The asymmetric unit contains one *N*-methyl-*N*-iso­propyl­tryptammonium (C_14_H_21_N_2_^+^) cation and one 3-carb­oxy­acrylate (C_4_H_3_O_4_^−^) anion. The indole ring system of the cation is near planar with an r.m.s. deviation from planarity of 0.006 Å. The singly protonated fumarate anion is in the *trans* configuration and is slightly distorted from planarity with an r.m.s. deviation of 0.133 Å and a carboxyl­ate twist angle of 18.370 (5)°. The *N*-methyl-*N*-iso­propyl­ammonium group is disordered over two orientations in a 0.630 (3):0.370 (3) ratio.

The mol­ecular structure of 4-HO-MiPT fumarate monohydrate is shown on the right of Fig. 1 ▸. The asymmetric unit contains one 4-hy­droxy-*N*-methyl-*N*-iso­propyl­trypt­ammo­nium (C_14_H_21_N_2_O^+^) cation, one 3-carb­oxy­acrylate anion and one water mol­ecule of crystallization. The indole ring system of the cation is close to planar with an r.m.s. deviation of 0.021 Å. The singly protonated fumarate anion is also near planar with an r.m.s. deviation of 0.049 Å. The *N*-methyl-*N*-iso­propyl­ammonium group shows a similar disorder to the MiPT structure over two orientations in a 0.775 (5):0.225 (5) ratio.

## Supra­molecular features   

**Figure 2:**
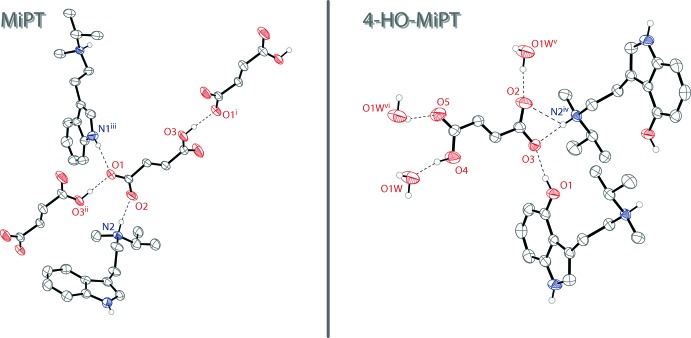
The hydrogen bonding of the fumarate ion in the structure of MiPT (left) and HO-MiPT (right). Displacement ellipsoids are drawn at the 50% probability level. Hydrogen atoms not involved in hydrogen bonds are omitted for clarity. Only one component of the amine disorder is shown. Symmetry codes: (i) *x*, 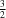 − *y*, −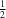 + *z* (ii) *x*, 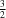 − *y*, 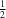 + *z* (iii) −1 + *x*, 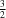 − *y*, −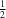 + *z* (iv) 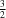 − *x*, 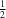 − *y*, 1 − *z* (v) *x*, −1 + *y*, *z* (vi) 1 − *x*, 2 − *y*, −*z*.

**Figure 3:**
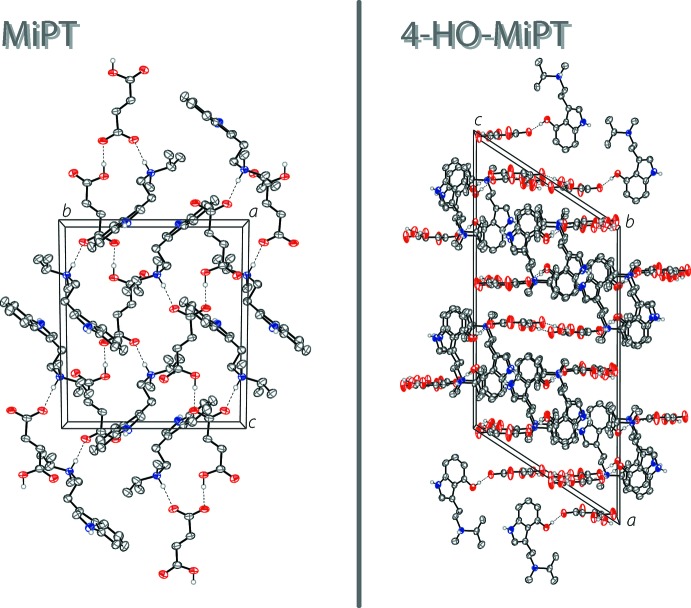
The crystal packing of MiPT fumarate (left), viewed along the *a* axis, and the crystal packing of HO-MiPT fumarate (right), viewed along the *b* axis. The hydrogen bonds (Tables 1 ▸ and 2 ▸) are shown as dashed lines. Displacement ellipsoids are drawn at the 50% probability level. Hydrogen atoms not involved in hydrogen bonds are omitted for clarity. Only one component of the amine disorder is shown.

In the extended structure of MiPT fumarate, the *N*-methyl-*N*-iso­propyl­amine and fumarate ions are linked into infinite two-dimensional networks lying parallel to the (010) plane through N—H⋯O and O—H⋯O hydrogen bonds (Table 1 ▸). The proton of the ammonium cation forms a hydrogen bond with one of the oxygen atoms of the deprotonated –CO_2_^−^ group of the 3-carb­oxy­acrylate ion. The carb­oxy­lic acid proton forms a hydrogen bond with an oxygen atom of an adjacent 3-carb­oxy­acrylate anion. The N—H grouping of the indole ring also hydrogen bonds to one of the oxygen atoms of the 3-carb­oxy­acrylate anion. The hydrogen bonding is shown on the left in Fig. 2 ▸, and the packing of MiPT fumarate is shown on the left in Fig. 3 ▸.

In the structure of 4-HO-MiPT fumarate, there are N—H⋯O and O—H⋯O hydrogen bonds that link together the cations and anions as well as the water mol­ecules of crystallization (Table 2 ▸). The result is a two-dimensional network lying parallel to the (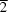
01) plane. The proton of the ammonium cation forms a bifurcated N—H⋯(O,O) hydrogen bond with the deprotonated –CO_2_^−^ group of the 3-carb­oxy­acrylate ion. The hydrogen of the hy­droxy group also hydrogen bonds to the same oxygen atom of the anion. The carb­oxy­lic acid proton hydrogen bonds with a water mol­ecule in the structure. Two other water mol­ecules form hydrogen bonds with two different oxygen atoms of the anion. The hydrogen bonding is shown on the right in Fig. 2 ▸, and the packing of 4-HO-MiPT fumarate is shown on the right in Fig. 3 ▸.

## Database survey   

The MiPT structure described above is a derivative of DMT (*N*,*N*-di­methyl­tryptamine), which has been structurally characterized (Falkenberg, 1972 ▸), as well as its close derivative MPT, *N*-methyl-*N*-propyl­tryptamine (Chadeayne *et al.* 2019*b* ▸). In both cases, these were crystallized as free bases, while MiPT is the fumarate salt. In the case of 4-HO-MiPT, the most closely related mol­ecule is psilocin, which has been structurally characterized (Petcher & Weber, 1974 ▸), as well as psilocybin (Weber & Petcher, 1974 ▸). Psilocin was reported as the free base and psilocybin was reported as a zwitterionic mol­ecule, while the structure of 4-HO-MiPT reported here is the hydrated fumarate salt. Two different ionic structures of the 4-acet­oxy derivative of DMT have been reported as fumarate salts (Chadeayne *et al.* 2019*a* ▸,*c* ▸). The metrical parameters of the tryptammonium cations for MiPT and 4-HO-MiPT are consistent with those of the other tryptammonium structures reported.

## Synthesis and crystallization   

Single crystals suitable for X-ray analysis were obtained from the slow evaporation of aqueous solutions of commercial samples of *N*-methyl-*N*-iso­propyl­tryptammonium fumarate and 4-hy­droxy-*N*-methyl-*N*-iso­propyl­tryptammonium fumarate (The Indole Shop).

## Refinement   

Crystal data, data collection and structure refinement details are summarized in Table 3 ▸. C-bound H atoms were placed in calculated positions (C—H = 0.95–1.00 Å) and refined as riding with *U*_iso_(H) = 1.2*U*_eq_(C) or 1.5*U*_eq_(C-meth­yl). The following restraints were applied: C—N = 1.54±0.01, N—H = 0.87±0.01, O—H = 0.86±0.01 Å. N- and O-bound H atoms were refined with *U*_iso_(H) = 1.5*U*_eq_(N,O). In the MiPT fumarate, the *N*-methyl-*N*-iso­propyl­aminium group is disordered. It is modeled as two components: N2 and C11–C14 with an occupancy of 0.630 (3) and N2*A* and C11*A*–C14*A* with an occupancy of 0.370 (3). 4-HO MiPT fumarate exhibits a similar disorder of the *N*-methyl-*N*-iso­propyl­aminium group that is modeled as two components: N2, C11*-*-C14 with an occupancy of 0.775 (5) and N2*A*, C11*A*–C14*A* with an occupancy of 0.225 (5).

## Supplementary Material

Crystal structure: contains datablock(s) MiPT, 4-HO-MiPT, I. DOI: 10.1107/S2056989019011253/hb7844sup1.cif

Structure factors: contains datablock(s) MiPT. DOI: 10.1107/S2056989019011253/hb7844MiPTsup2.hkl

Structure factors: contains datablock(s) 4-HO-MiPT. DOI: 10.1107/S2056989019011253/hb78444-HO-MiPTsup3.hkl

Supporting information file. DOI: 10.1107/S2056989019011253/hb7844MiPTsup4.cml

Supporting information file. DOI: 10.1107/S2056989019011253/hb78444-HO-MiPTsup5.cml

CCDC references: 1946718, 1946717

Additional supporting information:  crystallographic information; 3D view; checkCIF report

## Figures and Tables

**Table 1:** Hydrogen-bond geometry (Å, °) for MiPT[Chem scheme1]

*D*—H⋯*A*	*D*—H	H⋯*A*	*D*⋯*A*	*D*—H⋯*A*
O3—H3*A*⋯O1^i^	0.87 (1)	1.66 (1)	2.5316 (18)	176 (3)
N1—H1⋯O1^ii^	0.87 (1)	2.04 (1)	2.874 (2)	160 (2)
N2—H2⋯O2	0.88 (1)	1.79 (1)	2.667 (3)	173 (3)
N2*A*—H2*A*⋯O2	0.88 (1)	1.81 (2)	2.670 (5)	167 (6)

Symmetry codes: (i) 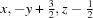; (ii) 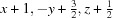.

**Table 2:** Hydrogen-bond geometry (Å, °) for 4-HO-MiPT[Chem scheme1]

*D*—H⋯*A*	*D*—H	H⋯*A*	*D*⋯*A*	*D*—H⋯*A*
N2—H2⋯O2^i^	0.88 (1)	2.51 (2)	3.085 (2)	124 (2)
N2—H2⋯O3^i^	0.88 (1)	1.89 (1)	2.775 (2)	178 (2)
N2*A*—H2*A*⋯O3^i^	0.87 (1)	1.85 (2)	2.717 (6)	172 (8)
O1—H1⋯O3	0.87 (2)	1.79 (3)	2.6512 (17)	172 (2)
O4—H4*A*⋯O1*W*	0.93 (3)	1.66 (3)	2.579 (2)	167 (3)
O1*W*—H1*WA*⋯O5^ii^	0.84 (3)	1.98 (3)	2.779 (2)	160 (2)
O1*W*—H1*WB*⋯O2^iii^	0.87 (3)	1.74 (3)	2.599 (2)	170 (2)

Symmetry codes: (i) 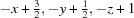; (ii) 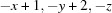; (iii) 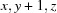.

**Table 3:** Experimental details

	MiPT	4-HO-MiPT
Crystal data		
Chemical formula	C_14_H_21_N_2_^+^·C_4_H_3_O_4_^−^	C_14_H_21_N_2_O^+^·C_4_H_3_O_4_^−^·H_2_O
*M*_r_	332.39	366.41
Crystal system, space group	Monoclinic, *P*2_1_/*c*	Monoclinic, *C*2/*c*
Temperature (K)	200	200
*a*, *b*, *c* (Å)	9.852 (2), 12.789 (2), 14.875 (3)	29.507 (3), 8.7445 (8), 17.3659 (18)
β (°)	106.932 (7)	123.389 (3)
*V* (Å^3^)	1793.0 (6)	3741.2 (7)
*Z*	4	8
Radiation type	Mo *K*α	Mo *K*α
μ (mm^−1^)	0.09	0.10
Crystal size (mm)	0.20 × 0.18 × 0.05	0.30 × 0.25 × 0.20

Data collection		
Diffractometer	Bruker D8 Venture CMOS	Bruker D8 Venture CMOS
Absorption correction	Multi-scan (*SADABS*; Bruker, 2016 ▸)	Multi-scan (*SADABS*; Bruker, 2016 ▸)
*T*_min_, *T*_max_	0.687, 0.745	0.719, 0.745
No. of measured, independent and observed [*I* > 2σ(*I*)] reflections	36899, 3297, 2605	70395, 3458, 2978
*R*_int_	0.052	0.041
(sin θ/λ)_max_ (Å^−1^)	0.604	0.604

Refinement		
*R*[*F* ^2^ > 2σ(*F* ^2^)], *wR*(*F* ^2^), *S*	0.049, 0.127, 1.06	0.041, 0.096, 1.08
No. of reflections	3297	3458
No. of parameters	240	320
No. of restraints	8	12
H-atom treatment	H atoms treated by a mixture of independent and constrained refinement	H atoms treated by a mixture of independent and constrained refinement
Δρ_max_, Δρ_min_ (e Å^−3^)	0.26, −0.26	0.22, −0.20

Computer programs: *APEX3* and *SAINT* (Bruker, 2016 ▸), *SHELXS97* and *SHELXL97* (Sheldrick, 2008 ▸) and *OLEX2* (Dolomanov *et al.*, 2009 ▸).

## supplementary crystallographic information

### [2-(1H-Indol-3-yl)ethyl](methyl)propan-2-ylazanium 3-carboxyprop-2-enoate (MiPT) Crystal data

**Table:**

C_14_H_21_N_2_^+^·C_4_H_3_O_4_^−^	*F*(000) = 712
*M**_r_* = 332.39	*D*_x_ = 1.231 Mg m^−^^3^
Monoclinic, *P*2_1_/*c*	Mo *K*α radiation, λ = 0.71073 Å
*a* = 9.852 (2) Å	Cell parameters from 9752 reflections
*b* = 12.789 (2) Å	θ = 3.0–25.3°
*c* = 14.875 (3) Å	µ = 0.09 mm^−^^1^
β = 106.932 (7)°	*T* = 200 K
*V* = 1793.0 (6) Å^3^	BLOCK, colourless
*Z* = 4	0.20 × 0.18 × 0.05 mm

### [2-(1H-Indol-3-yl)ethyl](methyl)propan-2-ylazanium 3-carboxyprop-2-enoate (MiPT) Data collection

**Table:**

Bruker D8 Venture CMOS diffractometer	2605 reflections with *I* > 2σ(*I*)
φ and ω scans	*R*_int_ = 0.052
Absorption correction: multi-scan (SADABS; Bruker, 2016)	θ_max_ = 25.4°, θ_min_ = 3.0°
*T*_min_ = 0.687, *T*_max_ = 0.745	*h* = −11→11
36899 measured reflections	*k* = −15→15
3297 independent reflections	*l* = −17→17

### [2-(1H-Indol-3-yl)ethyl](methyl)propan-2-ylazanium 3-carboxyprop-2-enoate (MiPT) Refinement

**Table:**

Refinement on *F*^2^	Hydrogen site location: mixed
Least-squares matrix: full	H atoms treated by a mixture of independent and constrained refinement
*R*[*F*^2^ > 2σ(*F*^2^)] = 0.049	*w* = 1/[σ^2^(*F*_o_^2^) + (0.0488*P*)^2^ + 1.0971*P*] where *P* = (*F*_o_^2^ + 2*F*_c_^2^)/3
*wR*(*F*^2^) = 0.127	(Δ/σ)_max_ < 0.001
*S* = 1.06	Δρ_max_ = 0.26 e Å^−^^3^
3297 reflections	Δρ_min_ = −0.25 e Å^−^^3^
240 parameters	Extinction correction: SHELXL, Fc^*^=kFc[1+0.001xFc^2^λ^3^/sin(2θ)]^-1/4^
8 restraints	Extinction coefficient: 0.039 (3)

### [2-(1H-Indol-3-yl)ethyl](methyl)propan-2-ylazanium 3-carboxyprop-2-enoate (MiPT) Special details

**Table:**

Geometry. All esds (except the esd in the dihedral angle between two l.s. planes) are estimated using the full covariance matrix. The cell esds are taken into account individually in the estimation of esds in distances, angles and torsion angles; correlations between esds in cell parameters are only used when they are defined by crystal symmetry. An approximate (isotropic) treatment of cell esds is used for estimating esds involving l.s. planes.

### [2-(1H-Indol-3-yl)ethyl](methyl)propan-2-ylazanium 3-carboxyprop-2-enoate (MiPT) Fractional atomic coordinates and isotropic or equivalent isotropic displacement parameters (Å^2^)

**Table:**

	*x*	*y*	*z*	*U*_iso_*/*U*_eq_	Occ. (<1)
O1	0.17442 (15)	0.78060 (11)	0.58471 (8)	0.0395 (4)	
O2	0.21577 (18)	0.61007 (12)	0.58250 (9)	0.0521 (4)	
O3	0.19484 (17)	0.72434 (11)	0.25829 (9)	0.0442 (4)	
H3A	0.184 (3)	0.724 (2)	0.1982 (8)	0.066*	
O4	0.1592 (2)	0.55393 (12)	0.24156 (11)	0.0731 (6)	
N1	0.88917 (17)	0.64452 (13)	1.00719 (12)	0.0382 (4)	
H1	0.9809 (11)	0.6526 (17)	1.0259 (15)	0.046*	
N2	0.3185 (3)	0.5163 (2)	0.74864 (18)	0.0303 (7)	0.630 (3)
H2	0.281 (3)	0.551 (2)	0.6962 (14)	0.036*	0.630 (3)
C11	0.3410 (4)	0.4055 (3)	0.7190 (2)	0.0443 (4)	0.630 (3)
H11	0.3829	0.3640	0.7775	0.053*	0.630 (3)
C12	0.2020 (4)	0.3543 (3)	0.6666 (3)	0.0443 (4)	0.630 (3)
H12A	0.2210	0.2905	0.6359	0.066*	0.630 (3)
H12B	0.1459	0.4027	0.6191	0.066*	0.630 (3)
H12C	0.1490	0.3365	0.7110	0.066*	0.630 (3)
C13	0.4553 (5)	0.4179 (4)	0.6694 (3)	0.0443 (4)	0.630 (3)
H13A	0.5401	0.4493	0.7127	0.066*	0.630 (3)
H13B	0.4200	0.4631	0.6143	0.066*	0.630 (3)
H13C	0.4794	0.3491	0.6493	0.066*	0.630 (3)
C14	0.2176 (6)	0.5221 (4)	0.8107 (4)	0.0443 (4)	0.630 (3)
H14A	0.2112	0.5946	0.8304	0.066*	0.630 (3)
H14B	0.2545	0.4779	0.8664	0.066*	0.630 (3)
H14C	0.1232	0.4974	0.7749	0.066*	0.630 (3)
N2A	0.3757 (5)	0.4914 (4)	0.7207 (3)	0.0342 (11)	0.370 (3)
H2A	0.336 (6)	0.533 (4)	0.674 (3)	0.041*	0.370 (3)
C11A	0.2757 (6)	0.4302 (5)	0.7616 (4)	0.0443 (4)	0.370 (3)
H11A	0.3302	0.3822	0.8124	0.053*	0.370 (3)
C12A	0.1685 (8)	0.3698 (6)	0.6867 (5)	0.0443 (4)	0.370 (3)
H12D	0.2131	0.3066	0.6710	0.066*	0.370 (3)
H12E	0.1341	0.4134	0.6305	0.066*	0.370 (3)
H12F	0.0887	0.3500	0.7099	0.066*	0.370 (3)
C13A	0.4250 (8)	0.4028 (6)	0.6507 (5)	0.0443 (4)	0.370 (3)
H13D	0.5166	0.4234	0.6426	0.066*	0.370 (3)
H13E	0.3532	0.4006	0.5892	0.066*	0.370 (3)
H13F	0.4336	0.3335	0.6800	0.066*	0.370 (3)
C14A	0.1952 (10)	0.5037 (7)	0.7980 (7)	0.0443 (4)	0.370 (3)
H14D	0.1334	0.4659	0.8279	0.066*	0.370 (3)
H14E	0.1373	0.5467	0.7465	0.066*	0.370 (3)
H14F	0.2601	0.5487	0.8445	0.066*	0.370 (3)
C1	0.8135 (2)	0.57049 (15)	0.94651 (13)	0.0380 (5)	
H1A	0.8536	0.5193	0.9156	0.046*	
C2	0.7970 (2)	0.70501 (14)	1.03799 (13)	0.0333 (4)	
C3	0.8232 (2)	0.78851 (15)	1.10072 (14)	0.0411 (5)	
H3	0.9169	0.8130	1.1293	0.049*	
C4	0.7083 (2)	0.83418 (16)	1.11972 (16)	0.0475 (5)	
H4	0.7230	0.8911	1.1625	0.057*	
C5	0.5702 (2)	0.79874 (17)	1.07743 (16)	0.0468 (5)	
H5	0.4929	0.8323	1.0916	0.056*	
C6	0.5444 (2)	0.71618 (15)	1.01564 (14)	0.0392 (5)	
H6	0.4501	0.6926	0.9874	0.047*	
C7	0.6584 (2)	0.66727 (14)	0.99481 (12)	0.0317 (4)	
C8	0.6725 (2)	0.58089 (14)	0.93698 (12)	0.0343 (4)	
C9	0.5561 (2)	0.51227 (15)	0.87944 (13)	0.0410 (5)	
H9A	0.4972	0.4877	0.9190	0.049*	
H9B	0.5982	0.4502	0.8581	0.049*	
C10	0.4622 (2)	0.56961 (15)	0.79425 (13)	0.0413 (5)	
H10A	0.5141	0.5759	0.7466	0.050*	0.630 (3)
H10B	0.4446	0.6412	0.8135	0.050*	0.630 (3)
H10C	0.5221	0.6129	0.7657	0.050*	0.370 (3)
H10D	0.3967	0.6167	0.8143	0.050*	0.370 (3)
C15	0.19062 (19)	0.69693 (15)	0.54455 (12)	0.0311 (4)	
C16	0.17850 (19)	0.70575 (15)	0.44231 (12)	0.0314 (4)	
H16	0.1595	0.7725	0.4133	0.038*	
C17	0.1928 (2)	0.62619 (16)	0.39115 (13)	0.0379 (5)	
H17	0.2124	0.5600	0.4212	0.045*	
C18	0.1807 (2)	0.63118 (15)	0.28967 (13)	0.0366 (5)	

### [2-(1H-Indol-3-yl)ethyl](methyl)propan-2-ylazanium 3-carboxyprop-2-enoate (MiPT) Atomic displacement parameters (Å^2^)

**Table:**

	*U*^11^	*U*^22^	*U*^33^	*U*^12^	*U*^13^	*U*^23^
O1	0.0506 (8)	0.0474 (8)	0.0207 (6)	0.0070 (6)	0.0106 (6)	−0.0012 (6)
O2	0.0820 (12)	0.0477 (9)	0.0241 (7)	0.0098 (8)	0.0117 (7)	0.0083 (6)
O3	0.0714 (10)	0.0420 (8)	0.0197 (7)	−0.0031 (7)	0.0143 (7)	−0.0007 (6)
O4	0.1495 (19)	0.0411 (9)	0.0324 (8)	−0.0018 (10)	0.0323 (10)	−0.0061 (7)
N1	0.0326 (9)	0.0421 (9)	0.0379 (9)	−0.0025 (7)	0.0068 (7)	−0.0066 (7)
N2	0.0357 (16)	0.0298 (14)	0.0220 (14)	−0.0031 (12)	0.0034 (12)	−0.0004 (11)
C11	0.0445 (12)	0.0460 (10)	0.0386 (10)	−0.0045 (7)	0.0064 (7)	−0.0135 (8)
C12	0.0445 (12)	0.0460 (10)	0.0386 (10)	−0.0045 (7)	0.0064 (7)	−0.0135 (8)
C13	0.0445 (12)	0.0460 (10)	0.0386 (10)	−0.0045 (7)	0.0064 (7)	−0.0135 (8)
C14	0.0445 (12)	0.0460 (10)	0.0386 (10)	−0.0045 (7)	0.0064 (7)	−0.0135 (8)
N2A	0.040 (3)	0.033 (3)	0.025 (2)	−0.002 (2)	0.002 (2)	0.000 (2)
C11A	0.0445 (12)	0.0460 (10)	0.0386 (10)	−0.0045 (7)	0.0064 (7)	−0.0135 (8)
C12A	0.0445 (12)	0.0460 (10)	0.0386 (10)	−0.0045 (7)	0.0064 (7)	−0.0135 (8)
C13A	0.0445 (12)	0.0460 (10)	0.0386 (10)	−0.0045 (7)	0.0064 (7)	−0.0135 (8)
C14A	0.0445 (12)	0.0460 (10)	0.0386 (10)	−0.0045 (7)	0.0064 (7)	−0.0135 (8)
C1	0.0453 (12)	0.0365 (10)	0.0305 (10)	0.0001 (9)	0.0084 (9)	−0.0056 (8)
C2	0.0369 (10)	0.0314 (9)	0.0296 (9)	−0.0035 (8)	0.0066 (8)	−0.0005 (7)
C3	0.0431 (11)	0.0376 (11)	0.0385 (11)	−0.0104 (9)	0.0055 (9)	−0.0081 (9)
C4	0.0602 (14)	0.0360 (11)	0.0467 (12)	−0.0029 (10)	0.0159 (11)	−0.0117 (9)
C5	0.0477 (13)	0.0433 (12)	0.0514 (13)	0.0043 (10)	0.0175 (10)	−0.0052 (10)
C6	0.0346 (10)	0.0393 (11)	0.0409 (11)	−0.0028 (8)	0.0069 (9)	0.0015 (9)
C7	0.0360 (10)	0.0288 (9)	0.0264 (9)	−0.0031 (7)	0.0029 (8)	0.0020 (7)
C8	0.0419 (11)	0.0313 (9)	0.0252 (9)	−0.0041 (8)	0.0025 (8)	−0.0006 (7)
C9	0.0505 (12)	0.0342 (10)	0.0288 (10)	−0.0102 (9)	−0.0033 (9)	0.0004 (8)
C10	0.0500 (12)	0.0360 (10)	0.0291 (10)	−0.0150 (9)	−0.0025 (9)	0.0034 (8)
C15	0.0283 (9)	0.0436 (11)	0.0201 (9)	0.0028 (8)	0.0049 (7)	0.0025 (8)
C16	0.0340 (10)	0.0384 (10)	0.0208 (9)	0.0026 (8)	0.0063 (7)	0.0036 (7)
C17	0.0505 (12)	0.0392 (10)	0.0237 (9)	0.0066 (9)	0.0104 (8)	0.0038 (8)
C18	0.0478 (12)	0.0392 (11)	0.0232 (9)	0.0067 (9)	0.0108 (8)	−0.0008 (8)

### [2-(1H-Indol-3-yl)ethyl](methyl)propan-2-ylazanium 3-carboxyprop-2-enoate (MiPT) Geometric parameters (Å, º)

**Table:**

O1—C15	1.258 (2)	C12A—H12F	0.9800
O2—C15	1.238 (2)	C13A—H13D	0.9800
O3—H3A	0.869 (10)	C13A—H13E	0.9800
O3—C18	1.302 (2)	C13A—H13F	0.9800
O4—C18	1.202 (2)	C14A—H14D	0.9800
N1—H1	0.870 (10)	C14A—H14E	0.9800
N1—C1	1.369 (2)	C14A—H14F	0.9800
N1—C2	1.369 (3)	C1—H1A	0.9500
N2—H2	0.880 (10)	C1—C8	1.361 (3)
N2—C11	1.518 (4)	C2—C3	1.392 (3)
N2—C14	1.543 (7)	C2—C7	1.414 (3)
N2—C10	1.540 (3)	C3—H3	0.9500
C11—H11	1.0000	C3—C4	1.375 (3)
C11—C12	1.513 (5)	C4—H4	0.9500
C11—C13	1.523 (6)	C4—C5	1.397 (3)
C12—H12A	0.9800	C5—H5	0.9500
C12—H12B	0.9800	C5—C6	1.374 (3)
C12—H12C	0.9800	C6—H6	0.9500
C13—H13A	0.9800	C6—C7	1.397 (3)
C13—H13B	0.9800	C7—C8	1.432 (3)
C13—H13C	0.9800	C8—C9	1.499 (3)
C14—H14A	0.9800	C9—H9A	0.9900
C14—H14B	0.9800	C9—H9B	0.9900
C14—H14C	0.9800	C9—C10	1.522 (3)
N2A—H2A	0.876 (10)	C10—H10A	0.9900
N2A—C11A	1.518 (6)	C10—H10B	0.9900
N2A—C13A	1.702 (10)	C10—H10C	0.9900
N2A—C10	1.543 (4)	C10—H10D	0.9900
C11A—H11A	1.0000	C15—C16	1.495 (2)
C11A—C12A	1.506 (9)	C16—H16	0.9500
C11A—C14A	1.433 (12)	C16—C17	1.303 (3)
C12A—H12D	0.9800	C17—H17	0.9500
C12A—H12E	0.9800	C17—C18	1.481 (3)
			
C18—O3—H3A	111.8 (17)	C11A—C14A—H14E	109.5
C1—N1—H1	127.4 (15)	C11A—C14A—H14F	109.5
C2—N1—H1	123.6 (15)	H14D—C14A—H14E	109.5
C2—N1—C1	108.93 (16)	H14D—C14A—H14F	109.5
C11—N2—H2	106 (2)	H14E—C14A—H14F	109.5
C11—N2—C14	113.1 (3)	N1—C1—H1A	124.9
C11—N2—C10	110.3 (3)	C8—C1—N1	110.14 (17)
C14—N2—H2	109 (2)	C8—C1—H1A	124.9
C10—N2—H2	105 (2)	N1—C2—C3	130.27 (18)
C10—N2—C14	112.6 (2)	N1—C2—C7	107.58 (16)
N2—C11—H11	107.5	C3—C2—C7	122.15 (18)
N2—C11—C13	103.6 (3)	C2—C3—H3	121.3
C12—C11—N2	111.5 (3)	C4—C3—C2	117.45 (19)
C12—C11—H11	107.5	C4—C3—H3	121.3
C12—C11—C13	118.8 (3)	C3—C4—H4	119.3
C13—C11—H11	107.5	C3—C4—C5	121.44 (19)
C11—C12—H12A	109.5	C5—C4—H4	119.3
C11—C12—H12B	109.5	C4—C5—H5	119.4
C11—C12—H12C	109.5	C6—C5—C4	121.1 (2)
H12A—C12—H12B	109.5	C6—C5—H5	119.4
H12A—C12—H12C	109.5	C5—C6—H6	120.4
H12B—C12—H12C	109.5	C5—C6—C7	119.22 (19)
C11—C13—H13A	109.5	C7—C6—H6	120.4
C11—C13—H13B	109.5	C2—C7—C8	106.62 (17)
C11—C13—H13C	109.5	C6—C7—C2	118.61 (17)
H13A—C13—H13B	109.5	C6—C7—C8	134.76 (18)
H13A—C13—H13C	109.5	C1—C8—C7	106.73 (16)
H13B—C13—H13C	109.5	C1—C8—C9	126.19 (18)
N2—C14—H14A	109.5	C7—C8—C9	127.05 (18)
N2—C14—H14B	109.5	C8—C9—H9A	109.3
N2—C14—H14C	109.5	C8—C9—H9B	109.3
H14A—C14—H14B	109.5	C8—C9—C10	111.81 (16)
H14A—C14—H14C	109.5	H9A—C9—H9B	107.9
H14B—C14—H14C	109.5	C10—C9—H9A	109.3
C11A—N2A—H2A	116 (4)	C10—C9—H9B	109.3
C11A—N2A—C13A	103.7 (4)	N2—C10—H10A	108.7
C11A—N2A—C10	109.8 (4)	N2—C10—H10B	108.7
C13A—N2A—H2A	94 (4)	N2A—C10—H10C	109.5
C10—N2A—H2A	101 (4)	N2A—C10—H10D	109.5
C10—N2A—C13A	131.9 (5)	C9—C10—N2	114.26 (17)
N2A—C11A—H11A	110.5	C9—C10—N2A	110.7 (2)
C12A—C11A—N2A	111.4 (5)	C9—C10—H10A	108.7
C12A—C11A—H11A	110.5	C9—C10—H10B	108.7
C14A—C11A—N2A	107.9 (6)	C9—C10—H10C	109.5
C14A—C11A—H11A	110.5	C9—C10—H10D	109.5
C14A—C11A—C12A	105.7 (5)	H10A—C10—H10B	107.6
C11A—C12A—H12D	109.5	H10C—C10—H10D	108.1
C11A—C12A—H12E	109.5	O1—C15—C16	115.80 (16)
C11A—C12A—H12F	109.5	O2—C15—O1	125.66 (16)
H12D—C12A—H12E	109.5	O2—C15—C16	118.53 (17)
H12D—C12A—H12F	109.5	C15—C16—H16	118.5
H12E—C12A—H12F	109.5	C17—C16—C15	123.04 (17)
N2A—C13A—H13D	109.5	C17—C16—H16	118.5
N2A—C13A—H13E	109.5	C16—C17—H17	117.6
N2A—C13A—H13F	109.5	C16—C17—C18	124.86 (18)
H13D—C13A—H13E	109.5	C18—C17—H17	117.6
H13D—C13A—H13F	109.5	O3—C18—C17	114.82 (16)
H13E—C13A—H13F	109.5	O4—C18—O3	123.88 (17)
C11A—C14A—H14D	109.5	O4—C18—C17	121.30 (18)
			
O1—C15—C16—C17	179.92 (19)	C2—C7—C8—C9	177.68 (18)
O2—C15—C16—C17	0.2 (3)	C3—C2—C7—C6	0.4 (3)
N1—C1—C8—C7	0.4 (2)	C3—C2—C7—C8	−178.93 (18)
N1—C1—C8—C9	−177.70 (17)	C3—C4—C5—C6	0.5 (3)
N1—C2—C3—C4	−179.1 (2)	C4—C5—C6—C7	−0.2 (3)
N1—C2—C7—C6	179.60 (16)	C5—C6—C7—C2	−0.3 (3)
N1—C2—C7—C8	0.2 (2)	C5—C6—C7—C8	178.9 (2)
C11—N2—C10—C9	−57.8 (3)	C6—C7—C8—C1	−179.6 (2)
C14—N2—C11—C12	57.6 (4)	C6—C7—C8—C9	−1.5 (3)
C14—N2—C11—C13	−173.6 (3)	C7—C2—C3—C4	−0.1 (3)
C14—N2—C10—C9	69.7 (3)	C7—C8—C9—C10	71.3 (3)
C11A—N2A—C10—C9	63.3 (5)	C8—C9—C10—N2	−163.3 (2)
C13A—N2A—C11A—C12A	−47.0 (6)	C8—C9—C10—N2A	162.0 (3)
C13A—N2A—C11A—C14A	−162.6 (6)	C10—N2—C11—C12	−175.2 (3)
C13A—N2A—C10—C9	−68.0 (6)	C10—N2—C11—C13	−46.4 (3)
C1—N1—C2—C3	179.1 (2)	C10—N2A—C11A—C12A	168.1 (4)
C1—N1—C2—C7	0.0 (2)	C10—N2A—C11A—C14A	52.4 (6)
C1—C8—C9—C10	−111.0 (2)	C15—C16—C17—C18	179.57 (18)
C2—N1—C1—C8	−0.2 (2)	C16—C17—C18—O3	18.9 (3)
C2—C3—C4—C5	−0.3 (3)	C16—C17—C18—O4	−160.9 (2)
C2—C7—C8—C1	−0.4 (2)		

### [2-(1H-Indol-3-yl)ethyl](methyl)propan-2-ylazanium 3-carboxyprop-2-enoate (MiPT) Hydrogen-bond geometry (Å, º)

**Table:**

*D*—H···*A*	*D*—H	H···*A*	*D*···*A*	*D*—H···*A*
O3—H3*A*···O1^i^	0.87 (1)	1.66 (1)	2.5316 (18)	176 (3)
N1—H1···O1^ii^	0.87 (1)	2.04 (1)	2.874 (2)	160 (2)
N2—H2···O2	0.88 (1)	1.79 (1)	2.667 (3)	173 (3)
N2*A*—H2*A*···O2	0.88 (1)	1.81 (2)	2.670 (5)	167 (6)

Symmetry codes: (i) *x*, −*y*+3/2, *z*−1/2; (ii) *x*+1, −*y*+3/2, *z*+1/2.

### [2-(4-Hydroxy-1H-indol-3-yl)ethyl](methyl)propan-2-ylazanium 3-carboxyprop-2-enoate monohydrate (4-HO-MiPT) Crystal data

**Table:**

C_14_H_21_N_2_O^+^·C_4_H_3_O_4_^−^·H_2_O	*F*(000) = 1568
*M**_r_* = 366.41	*D*_x_ = 1.301 Mg m^−^^3^
Monoclinic, *C*2/*c*	Mo *K*α radiation, λ = 0.71073 Å
*a* = 29.507 (3) Å	Cell parameters from 9718 reflections
*b* = 8.7445 (8) Å	θ = 2.9–25.3°
*c* = 17.3659 (18) Å	µ = 0.10 mm^−^^1^
β = 123.389 (3)°	*T* = 200 K
*V* = 3741.2 (7) Å^3^	BLOCK, colourless
*Z* = 8	0.30 × 0.25 × 0.20 mm

### [2-(4-Hydroxy-1H-indol-3-yl)ethyl](methyl)propan-2-ylazanium 3-carboxyprop-2-enoate monohydrate (4-HO-MiPT) Data collection

**Table:**

Bruker D8 Venture CMOS diffractometer	2978 reflections with *I* > 2σ(*I*)
φ and ω scans	*R*_int_ = 0.041
Absorption correction: multi-scan (SADABS; Bruker, 2016)	θ_max_ = 25.4°, θ_min_ = 3.1°
*T*_min_ = 0.719, *T*_max_ = 0.745	*h* = −35→35
70395 measured reflections	*k* = −10→10
3458 independent reflections	*l* = −20→20

### [2-(4-Hydroxy-1H-indol-3-yl)ethyl](methyl)propan-2-ylazanium 3-carboxyprop-2-enoate monohydrate (4-HO-MiPT) Refinement

**Table:**

Refinement on *F*^2^	Hydrogen site location: mixed
Least-squares matrix: full	H atoms treated by a mixture of independent and constrained refinement
*R*[*F*^2^ > 2σ(*F*^2^)] = 0.041	*w* = 1/[σ^2^(*F*_o_^2^) + (0.0299*P*)^2^ + 3.9177*P*] where *P* = (*F*_o_^2^ + 2*F*_c_^2^)/3
*wR*(*F*^2^) = 0.096	(Δ/σ)_max_ < 0.001
*S* = 1.08	Δρ_max_ = 0.22 e Å^−^^3^
3458 reflections	Δρ_min_ = −0.20 e Å^−^^3^
320 parameters	Extinction correction: SHELXL, Fc^*^=kFc[1+0.001xFc^2^λ^3^/sin(2θ)]^-1/4^
12 restraints	Extinction coefficient: 0.0081 (4)

### [2-(4-Hydroxy-1H-indol-3-yl)ethyl](methyl)propan-2-ylazanium 3-carboxyprop-2-enoate monohydrate (4-HO-MiPT) Special details

**Table:**

Geometry. All esds (except the esd in the dihedral angle between two l.s. planes) are estimated using the full covariance matrix. The cell esds are taken into account individually in the estimation of esds in distances, angles and torsion angles; correlations between esds in cell parameters are only used when they are defined by crystal symmetry. An approximate (isotropic) treatment of cell esds is used for estimating esds involving l.s. planes.

### [2-(4-Hydroxy-1H-indol-3-yl)ethyl](methyl)propan-2-ylazanium 3-carboxyprop-2-enoate monohydrate (4-HO-MiPT) Fractional atomic coordinates and isotropic or equivalent isotropic displacement parameters (Å^2^)

**Table:**

	*x*	*y*	*z*	*U*_iso_*/*U*_eq_	Occ. (<1)
O1	0.69858 (5)	0.51214 (13)	0.50306 (8)	0.0369 (3)	
O1W	0.52724 (8)	1.07404 (17)	0.09877 (11)	0.0664 (5)	
O2	0.56158 (8)	0.24517 (15)	0.24314 (9)	0.0786 (6)	
O3	0.61162 (5)	0.35608 (13)	0.37801 (7)	0.0421 (3)	
O4	0.54759 (7)	0.80247 (15)	0.16809 (9)	0.0621 (4)	
O5	0.49277 (7)	0.70299 (16)	0.03041 (9)	0.0718 (5)	
N1	0.77812 (6)	0.83838 (17)	0.75125 (10)	0.0411 (4)	
C1	0.73684 (6)	0.67519 (16)	0.63265 (10)	0.0277 (3)	
C2	0.69254 (6)	0.58832 (17)	0.56519 (10)	0.0304 (3)	
C3	0.64541 (7)	0.58566 (19)	0.56435 (11)	0.0376 (4)	
H3	0.6153	0.5269	0.5191	0.045*	
C4	0.64171 (7)	0.6688 (2)	0.62958 (12)	0.0420 (4)	
H4	0.6088	0.6650	0.6274	0.050*	
C5	0.68368 (7)	0.7552 (2)	0.69635 (12)	0.0410 (4)	
H5	0.6807	0.8105	0.7405	0.049*	
C6	0.73117 (7)	0.75834 (18)	0.69665 (10)	0.0337 (4)	
C7	0.81267 (7)	0.81061 (19)	0.72352 (11)	0.0376 (4)	
H7	0.8477	0.8547	0.7507	0.045*	
C8	0.78980 (6)	0.71090 (17)	0.65144 (10)	0.0303 (3)	
C9	0.81461 (7)	0.65950 (17)	0.60004 (11)	0.0327 (4)	
H9A	0.8449	0.7289	0.6150	0.039*	
H9B	0.7871	0.6673	0.5330	0.039*	
C10	0.83576 (7)	0.49632 (18)	0.62290 (11)	0.0319 (4)	
H10A	0.8586	0.4839	0.6907	0.038*	0.775 (5)
H10B	0.8046	0.4253	0.5984	0.038*	0.775 (5)
H10C	0.810 (3)	0.427 (9)	0.621 (5)	0.038*	0.225 (5)
H10D	0.868 (3)	0.486 (9)	0.684 (5)	0.038*	0.225 (5)
N2	0.86881 (10)	0.4538 (2)	0.58322 (14)	0.0299 (6)	0.775 (5)
H2	0.8759 (9)	0.3557 (13)	0.5963 (16)	0.036*	0.775 (5)
C11	0.83717 (10)	0.4648 (2)	0.47872 (14)	0.0347 (7)	0.775 (5)
H11	0.8309	0.5755	0.4615	0.042*	0.775 (5)
C12	0.8709 (2)	0.3979 (9)	0.4448 (4)	0.0500 (13)	0.775 (5)
H12A	0.9040	0.4584	0.4692	0.075*	0.775 (5)
H12B	0.8497	0.4003	0.3772	0.075*	0.775 (5)
H12C	0.8806	0.2919	0.4661	0.075*	0.775 (5)
C13	0.7823 (3)	0.3875 (9)	0.4349 (4)	0.0421 (12)	0.775 (5)
H13A	0.7633	0.3916	0.3676	0.063*	0.775 (5)
H13B	0.7607	0.4403	0.4539	0.063*	0.775 (5)
H13C	0.7874	0.2805	0.4549	0.063*	0.775 (5)
C14	0.92160 (18)	0.5366 (7)	0.6263 (3)	0.0410 (10)	0.775 (5)
H14A	0.9364	0.5538	0.6918	0.062*	0.775 (5)
H14B	0.9158	0.6352	0.5954	0.062*	0.775 (5)
H14C	0.9472	0.4752	0.6201	0.062*	0.775 (5)
N2A	0.8395 (3)	0.4167 (7)	0.5478 (5)	0.032 (2)	0.225 (5)
H2A	0.855 (3)	0.327 (4)	0.567 (5)	0.038*	0.225 (5)
C11A	0.8776 (3)	0.5046 (8)	0.5319 (6)	0.038 (2)	0.225 (5)
H11A	0.8584	0.5998	0.4974	0.046*	0.225 (5)
C12A	0.8893 (9)	0.410 (3)	0.4699 (16)	0.063 (6)	0.225 (5)
H12D	0.9164	0.4626	0.4635	0.094*	0.225 (5)
H12E	0.8558	0.3970	0.4089	0.094*	0.225 (5)
H12F	0.9033	0.3089	0.4978	0.094*	0.225 (5)
C13A	0.9288 (7)	0.554 (3)	0.6248 (13)	0.080 (8)	0.225 (5)
H13D	0.9558	0.5949	0.6140	0.120*	0.225 (5)
H13E	0.9440	0.4655	0.6660	0.120*	0.225 (5)
H13F	0.9190	0.6331	0.6532	0.120*	0.225 (5)
C14A	0.7888 (8)	0.371 (3)	0.4588 (10)	0.041 (4)	0.225 (5)
H14D	0.7606	0.3451	0.4699	0.061*	0.225 (5)
H14E	0.7963	0.2819	0.4332	0.061*	0.225 (5)
H14F	0.7764	0.4561	0.4150	0.061*	0.225 (5)
C15	0.57935 (7)	0.36013 (18)	0.29127 (11)	0.0373 (4)	
C16	0.56391 (6)	0.51453 (18)	0.24727 (11)	0.0348 (4)	
H16	0.5762	0.6017	0.2864	0.042*	
C17	0.53429 (7)	0.53709 (19)	0.15761 (11)	0.0380 (4)	
H17	0.5193	0.4499	0.1188	0.046*	
C18	0.52272 (7)	0.68853 (19)	0.11306 (11)	0.0378 (4)	
H1A	0.7846 (8)	0.902 (3)	0.7946 (15)	0.061 (6)*	
H1WA	0.5221 (10)	1.123 (3)	0.0534 (18)	0.075 (8)*	
H1WB	0.5371 (10)	1.140 (3)	0.1427 (18)	0.073 (7)*	
H1	0.6684 (10)	0.469 (3)	0.4607 (17)	0.076 (8)*	
H4A	0.5378 (13)	0.894 (4)	0.135 (2)	0.125 (11)*	

### [2-(4-Hydroxy-1H-indol-3-yl)ethyl](methyl)propan-2-ylazanium 3-carboxyprop-2-enoate monohydrate (4-HO-MiPT) Atomic displacement parameters (Å^2^)

**Table:**

	*U*^11^	*U*^22^	*U*^33^	*U*^12^	*U*^13^	*U*^23^
O1	0.0456 (7)	0.0314 (6)	0.0333 (6)	−0.0030 (5)	0.0214 (6)	−0.0072 (5)
O1W	0.1121 (14)	0.0383 (8)	0.0472 (9)	−0.0134 (8)	0.0428 (9)	−0.0065 (7)
O2	0.1293 (14)	0.0301 (7)	0.0322 (7)	0.0008 (8)	0.0165 (8)	−0.0033 (6)
O3	0.0499 (7)	0.0332 (6)	0.0275 (6)	0.0026 (5)	0.0113 (5)	0.0022 (5)
O4	0.0913 (11)	0.0326 (7)	0.0362 (7)	−0.0040 (7)	0.0184 (7)	0.0013 (6)
O5	0.0944 (11)	0.0447 (8)	0.0316 (7)	−0.0044 (8)	0.0063 (7)	0.0075 (6)
N1	0.0512 (9)	0.0411 (8)	0.0336 (7)	−0.0029 (7)	0.0251 (7)	−0.0114 (6)
C1	0.0364 (8)	0.0237 (7)	0.0244 (7)	0.0042 (6)	0.0176 (6)	0.0041 (6)
C2	0.0403 (9)	0.0228 (7)	0.0280 (7)	0.0045 (6)	0.0188 (7)	0.0062 (6)
C3	0.0365 (9)	0.0355 (9)	0.0369 (9)	0.0011 (7)	0.0178 (7)	0.0061 (7)
C4	0.0406 (9)	0.0463 (10)	0.0458 (10)	0.0100 (8)	0.0281 (8)	0.0130 (8)
C5	0.0519 (10)	0.0441 (10)	0.0381 (9)	0.0115 (8)	0.0318 (9)	0.0051 (8)
C6	0.0442 (9)	0.0309 (8)	0.0281 (8)	0.0057 (7)	0.0212 (7)	0.0028 (6)
C7	0.0390 (9)	0.0385 (9)	0.0351 (8)	−0.0027 (7)	0.0202 (7)	−0.0034 (7)
C8	0.0371 (8)	0.0264 (8)	0.0282 (7)	0.0030 (6)	0.0183 (7)	0.0022 (6)
C9	0.0404 (9)	0.0301 (8)	0.0328 (8)	0.0045 (7)	0.0234 (7)	0.0043 (6)
C10	0.0418 (9)	0.0312 (8)	0.0288 (8)	0.0047 (7)	0.0233 (7)	0.0045 (6)
N2	0.0369 (13)	0.0259 (10)	0.0279 (11)	0.0045 (9)	0.0186 (10)	0.0037 (8)
C11	0.0512 (17)	0.0285 (11)	0.0276 (12)	0.0075 (10)	0.0237 (12)	0.0049 (9)
C12	0.070 (3)	0.048 (2)	0.048 (3)	0.005 (2)	0.043 (3)	0.0000 (18)
C13	0.045 (2)	0.037 (2)	0.029 (2)	0.006 (2)	0.0113 (18)	0.001 (2)
C14	0.043 (2)	0.0354 (18)	0.048 (2)	−0.0057 (17)	0.0271 (19)	−0.0075 (14)
N2A	0.037 (4)	0.026 (4)	0.030 (4)	0.010 (3)	0.018 (4)	0.005 (3)
C11A	0.045 (5)	0.035 (4)	0.039 (5)	0.004 (4)	0.027 (5)	0.004 (3)
C12A	0.090 (15)	0.071 (10)	0.056 (12)	0.002 (11)	0.058 (12)	−0.009 (9)
C13A	0.039 (8)	0.090 (14)	0.068 (12)	−0.036 (7)	0.002 (8)	0.003 (10)
C14A	0.033 (6)	0.046 (8)	0.025 (8)	0.016 (5)	0.005 (5)	0.014 (7)
C15	0.0455 (9)	0.0306 (8)	0.0286 (8)	0.0031 (7)	0.0159 (7)	−0.0008 (7)
C16	0.0399 (9)	0.0279 (8)	0.0311 (8)	−0.0002 (7)	0.0161 (7)	−0.0021 (6)
C17	0.0415 (9)	0.0301 (8)	0.0303 (8)	0.0006 (7)	0.0121 (7)	−0.0014 (7)
C18	0.0395 (9)	0.0344 (9)	0.0293 (8)	0.0016 (7)	0.0125 (7)	0.0008 (7)

### [2-(4-Hydroxy-1H-indol-3-yl)ethyl](methyl)propan-2-ylazanium 3-carboxyprop-2-enoate monohydrate (4-HO-MiPT) Geometric parameters (Å, º)

**Table:**

O1—C2	1.3606 (18)	N2—C11	1.520 (3)
O1—H1	0.87 (2)	N2—C14	1.493 (5)
O1W—H1WA	0.84 (3)	C11—H11	1.0000
O1W—H1WB	0.87 (3)	C11—C12	1.524 (5)
O2—C15	1.225 (2)	C11—C13	1.518 (6)
O3—C15	1.2642 (19)	C12—H12A	0.9800
O4—C18	1.293 (2)	C12—H12B	0.9800
O4—H4A	0.93 (3)	C12—H12C	0.9800
O5—C18	1.209 (2)	C13—H13A	0.9800
N1—C6	1.365 (2)	C13—H13B	0.9800
N1—C7	1.368 (2)	C13—H13C	0.9800
N1—H1A	0.87 (2)	C14—H14A	0.9800
C1—C2	1.404 (2)	C14—H14B	0.9800
C1—C6	1.413 (2)	C14—H14C	0.9800
C1—C8	1.444 (2)	N2A—H2A	0.872 (10)
C2—C3	1.383 (2)	N2A—C11A	1.507 (8)
C3—H3	0.9500	N2A—C14A	1.497 (10)
C3—C4	1.400 (2)	C11A—H11A	1.0000
C4—H4	0.9500	C11A—C12A	1.543 (10)
C4—C5	1.367 (3)	C11A—C13A	1.545 (10)
C5—H5	0.9500	C12A—H12D	0.9800
C5—C6	1.398 (2)	C12A—H12E	0.9800
C7—H7	0.9500	C12A—H12F	0.9800
C7—C8	1.361 (2)	C13A—H13D	0.9800
C8—C9	1.501 (2)	C13A—H13E	0.9800
C9—H9A	0.9900	C13A—H13F	0.9800
C9—H9B	0.9900	C14A—H14D	0.9800
C9—C10	1.520 (2)	C14A—H14E	0.9800
C10—H10A	0.9900	C14A—H14F	0.9800
C10—H10B	0.9900	C15—C16	1.494 (2)
C10—H10C	0.97 (8)	C16—H16	0.9500
C10—H10D	0.96 (8)	C16—C17	1.315 (2)
C10—N2	1.518 (2)	C17—H17	0.9500
C10—N2A	1.536 (6)	C17—C18	1.476 (2)
N2—H2	0.882 (10)		
			
C2—O1—H1	111.3 (16)	C11—C12—H12A	109.5
H1WA—O1W—H1WB	106 (2)	C11—C12—H12B	109.5
C18—O4—H4A	110 (2)	C11—C12—H12C	109.5
C6—N1—C7	109.43 (14)	H12A—C12—H12B	109.5
C6—N1—H1A	125.7 (14)	H12A—C12—H12C	109.5
C7—N1—H1A	124.7 (14)	H12B—C12—H12C	109.5
C2—C1—C6	118.41 (14)	C11—C13—H13A	109.5
C2—C1—C8	134.44 (14)	C11—C13—H13B	109.5
C6—C1—C8	107.03 (13)	C11—C13—H13C	109.5
O1—C2—C1	117.29 (14)	H13A—C13—H13B	109.5
O1—C2—C3	123.58 (15)	H13A—C13—H13C	109.5
C3—C2—C1	119.13 (14)	H13B—C13—H13C	109.5
C2—C3—H3	119.7	N2—C14—H14A	109.5
C2—C3—C4	120.57 (16)	N2—C14—H14B	109.5
C4—C3—H3	119.7	N2—C14—H14C	109.5
C3—C4—H4	118.8	H14A—C14—H14B	109.5
C5—C4—C3	122.39 (16)	H14A—C14—H14C	109.5
C5—C4—H4	118.8	H14B—C14—H14C	109.5
C4—C5—H5	121.6	C10—N2A—H2A	110 (6)
C4—C5—C6	116.89 (15)	C11A—N2A—C10	109.5 (6)
C6—C5—H5	121.6	C11A—N2A—H2A	104 (6)
N1—C6—C1	107.22 (14)	C14A—N2A—C10	120.0 (13)
N1—C6—C5	130.14 (15)	C14A—N2A—H2A	100 (5)
C5—C6—C1	122.61 (15)	C14A—N2A—C11A	111.7 (11)
N1—C7—H7	124.8	N2A—C11A—H11A	107.5
C8—C7—N1	110.45 (15)	N2A—C11A—C12A	109.2 (12)
C8—C7—H7	124.8	N2A—C11A—C13A	110.6 (13)
C1—C8—C9	128.47 (14)	C12A—C11A—H11A	107.5
C7—C8—C1	105.86 (13)	C12A—C11A—C13A	114.4 (14)
C7—C8—C9	125.56 (15)	C13A—C11A—H11A	107.5
C8—C9—H9A	109.0	C11A—C12A—H12D	109.5
C8—C9—H9B	109.0	C11A—C12A—H12E	109.5
C8—C9—C10	112.98 (12)	C11A—C12A—H12F	109.5
H9A—C9—H9B	107.8	H12D—C12A—H12E	109.5
C10—C9—H9A	109.0	H12D—C12A—H12F	109.5
C10—C9—H9B	109.0	H12E—C12A—H12F	109.5
C9—C10—H10A	109.1	C11A—C13A—H13D	109.5
C9—C10—H10B	109.1	C11A—C13A—H13E	109.5
C9—C10—H10C	112 (4)	C11A—C13A—H13F	109.5
C9—C10—H10D	113 (5)	H13D—C13A—H13E	109.5
C9—C10—N2A	114.5 (3)	H13D—C13A—H13F	109.5
H10A—C10—H10B	107.8	H13E—C13A—H13F	109.5
H10C—C10—H10D	105 (6)	N2A—C14A—H14D	109.5
N2—C10—C9	112.69 (13)	N2A—C14A—H14E	109.5
N2—C10—H10A	109.1	N2A—C14A—H14F	109.5
N2—C10—H10B	109.1	H14D—C14A—H14E	109.5
N2A—C10—H10C	97 (4)	H14D—C14A—H14F	109.5
N2A—C10—H10D	114 (5)	H14E—C14A—H14F	109.5
C10—N2—H2	104.1 (17)	O2—C15—O3	123.28 (15)
C10—N2—C11	113.62 (18)	O2—C15—C16	119.80 (14)
C11—N2—H2	105.8 (16)	O3—C15—C16	116.91 (14)
C14—N2—C10	114.0 (2)	C15—C16—H16	118.0
C14—N2—H2	107.9 (16)	C17—C16—C15	123.93 (15)
C14—N2—C11	110.7 (3)	C17—C16—H16	118.0
N2—C11—H11	108.0	C16—C17—H17	117.7
N2—C11—C12	109.5 (3)	C16—C17—C18	124.51 (15)
C12—C11—H11	108.0	C18—C17—H17	117.7
C13—C11—N2	110.8 (3)	O4—C18—C17	115.49 (14)
C13—C11—H11	108.0	O5—C18—O4	122.93 (16)
C13—C11—C12	112.2 (4)	O5—C18—C17	121.56 (15)
			
O1—C2—C3—C4	−178.98 (14)	C7—C8—C9—C10	106.26 (18)
O2—C15—C16—C17	−4.2 (3)	C8—C1—C2—O1	3.2 (2)
O3—C15—C16—C17	174.39 (17)	C8—C1—C2—C3	−176.09 (16)
N1—C7—C8—C1	0.49 (18)	C8—C1—C6—N1	−0.47 (17)
N1—C7—C8—C9	176.96 (14)	C8—C1—C6—C5	177.70 (14)
C1—C2—C3—C4	0.2 (2)	C8—C9—C10—N2	−170.62 (16)
C1—C8—C9—C10	−78.1 (2)	C8—C9—C10—N2A	156.1 (4)
C2—C1—C6—N1	−177.00 (13)	C9—C10—N2—C11	−60.2 (2)
C2—C1—C6—C5	1.2 (2)	C9—C10—N2—C14	67.9 (3)
C2—C1—C8—C7	175.72 (16)	C9—C10—N2A—C11A	60.3 (6)
C2—C1—C8—C9	−0.6 (3)	C9—C10—N2A—C14A	−70.7 (12)
C2—C3—C4—C5	−0.1 (3)	C10—N2—C11—C12	−171.7 (4)
C3—C4—C5—C6	0.4 (2)	C10—N2—C11—C13	−47.3 (4)
C4—C5—C6—N1	176.71 (17)	C10—N2A—C11A—C12A	169.9 (12)
C4—C5—C6—C1	−1.0 (2)	C10—N2A—C11A—C13A	43.1 (13)
C6—N1—C7—C8	−0.81 (19)	C14—N2—C11—C12	58.5 (4)
C6—C1—C2—O1	178.51 (13)	C14—N2—C11—C13	−177.2 (4)
C6—C1—C2—C3	−0.7 (2)	C14A—N2A—C11A—C12A	−54.8 (18)
C6—C1—C8—C7	−0.01 (17)	C14A—N2A—C11A—C13A	178.4 (18)
C6—C1—C8—C9	−176.34 (14)	C15—C16—C17—C18	−174.40 (16)
C7—N1—C6—C1	0.78 (18)	C16—C17—C18—O4	5.5 (3)
C7—N1—C6—C5	−177.20 (16)	C16—C17—C18—O5	−175.90 (19)

### [2-(4-Hydroxy-1H-indol-3-yl)ethyl](methyl)propan-2-ylazanium 3-carboxyprop-2-enoate monohydrate (4-HO-MiPT) Hydrogen-bond geometry (Å, º)

**Table:**

*D*—H···*A*	*D*—H	H···*A*	*D*···*A*	*D*—H···*A*
N2—H2···O2^i^	0.88 (1)	2.51 (2)	3.085 (2)	124 (2)
N2—H2···O3^i^	0.88 (1)	1.89 (1)	2.775 (2)	178 (2)
N2*A*—H2*A*···O3^i^	0.87 (1)	1.85 (2)	2.717 (6)	172 (8)
O1—H1···O3	0.87 (2)	1.79 (3)	2.6512 (17)	172 (2)
O4—H4*A*···O1*W*	0.93 (3)	1.66 (3)	2.579 (2)	167 (3)
O1*W*—H1*WA*···O5^ii^	0.84 (3)	1.98 (3)	2.779 (2)	160 (2)
O1*W*—H1*WB*···O2^iii^	0.87 (3)	1.74 (3)	2.599 (2)	170 (2)

Symmetry codes: (i) −*x*+3/2, −*y*+1/2, −*z*+1; (ii) −*x*+1, −*y*+2, −*z*; (iii) *x*, *y*+1, *z*.
